# Artificial Intelligence Techniques: Analysis, Application, and Outcome in Dentistry—A Systematic Review

**DOI:** 10.1155/2021/9751564

**Published:** 2021-06-22

**Authors:** Naseer Ahmed, Maria Shakoor Abbasi, Filza Zuberi, Warisha Qamar, Mohamad Syahrizal Bin Halim, Afsheen Maqsood, Mohammad Khursheed Alam

**Affiliations:** ^1^Prosthodontics Unit, School of Dental Sciences, Health Campus, Universiti Sains Malaysia, 16150 Kubang Kerian, Kota Bharu, Kelantan, Malaysia; ^2^Department of Prosthodontics, Altamash Institute of Dental Medicine, Karachi 75500, Pakistan; ^3^Undergraduate Student Bachelor of Dental Surgery, Dow Dental College, Dow University of Health Sciences, Karachi 74200, Pakistan; ^4^Research Intern, Department of Prosthodontics, Altamash Institute of Dental Medicine, Karachi 75500, Pakistan; ^5^Conservative Dentistry Unit, School of Dental Sciences, Health Campus, Universiti Sains Malaysia, 16150 Kubang Kerian, Kota Bharu, Kelantan, Malaysia; ^6^Department of Oral Pathology, Bahria University Medical and Dental College, Karachi 75530, Pakistan; ^7^Department of Preventive Dentistry, College of Dentistry, Jouf University, Sakaka, Al Jouf, 72345, Saudi Arabia

## Abstract

**Objective:**

The objective of this systematic review was to investigate the quality and outcome of studies into artificial intelligence techniques, analysis, and effect in dentistry.

**Materials and Methods:**

Using the MeSH keywords: artificial intelligence (AI), dentistry, AI in dentistry, neural networks and dentistry, machine learning, AI dental imaging, and AI treatment recommendations and dentistry. Two investigators performed an electronic search in 5 databases: PubMed/MEDLINE (National Library of Medicine), Scopus (Elsevier), ScienceDirect databases (Elsevier), Web of Science (Clarivate Analytics), and the Cochrane Collaboration (Wiley). The English language articles reporting on AI in different dental specialties were screened for eligibility. Thirty-two full-text articles were selected and systematically analyzed according to a predefined inclusion criterion. These articles were analyzed as per a specific research question, and the relevant data based on article general characteristics, study and control groups, assessment methods, outcomes, and quality assessment were extracted.

**Results:**

The initial search identified 175 articles related to AI in dentistry based on the title and abstracts. The full text of 38 articles was assessed for eligibility to exclude studies not fulfilling the inclusion criteria. Six articles not related to AI in dentistry were excluded. Thirty-two articles were included in the systematic review. It was revealed that AI provides accurate patient management, dental diagnosis, prediction, and decision making. Artificial intelligence appeared as a reliable modality to enhance future implications in the various fields of dentistry, i.e., diagnostic dentistry, patient management, head and neck cancer, restorative dentistry, prosthetic dental sciences, orthodontics, radiology, and periodontics.

**Conclusion:**

The included studies describe that AI is a reliable tool to make dental care smooth, better, time-saving, and economical for practitioners. AI benefits them in fulfilling patient demand and expectations. The dentists can use AI to ensure quality treatment, better oral health care outcome, and achieve precision. AI can help to predict failures in clinical scenarios and depict reliable solutions. However, AI is increasing the scope of state-of-the-art models in dentistry but is still under development. Further studies are required to assess the clinical performance of AI techniques in dentistry.

## 1. Introduction

**Figure 1:**
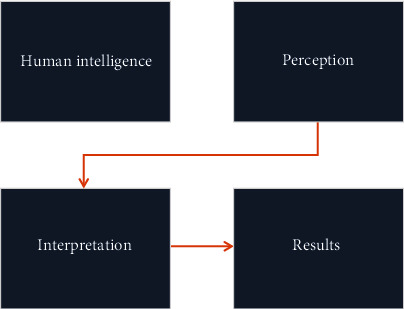
Schematic illustration of human intelligence networking.

**Figure 2:**
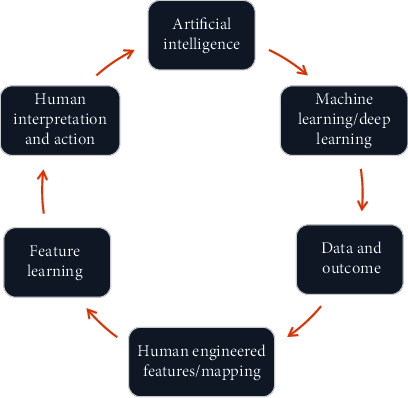
Schematic illustration of artificial intelligence model.

Artificial intelligence (AI) is a general term which refers to perform the task of human beings with the help of machine and technology. According to “Barr and Feigenbaum,” AI is the part of computer science concerned with designing an intelligent computer system that exhibits characteristics we associate with intelligence in human behavior-understanding language, learning, reasoning, problem solving, and many more [1]. There are subcategories of AI, which is machine learning and its allied fields like deep learning, cognitive computing, natural language processing, robotics, expert systems, and fuzzy logic. Machine learning is a subgroup of AI which enhances automated learning ability without being distinctly programmed. Its primary goal is to allow automated learning without human arbitration. AI models predict future events with the present set of observations [2]. The schematic presentation of AI and a human intelligence model is shown in Figures 1 and 2.

AI, similar to other fields, is transforming as an emerging field of dentistry. AI can perform a number of simple tasks in the dental clinic with greater precision, less staffing, and fewer errors than human counterparts; from booking and coordinating regular appointments to assisting the clinical diagnosis and treatment planning, AI can handle all [3]. The AI application showed high accuracy, sensitivity, specificity, and precision in detection and classification of malocclusion in orthodontics [4]. AI can automatically detect and classify dental restorations on panoramic radiographs along with assistance in the detection of dental and maxillofacial abnormalities such as periodontal diseases, root caries, bony lesions, i.e., BRONJ (bisphosphonate-related osteonecrosis of the jaw) associated with dental extraction, and facial defects [3, 5].

A popular field in machine learning is “deep learning,” where multilayered (deep) neural networks are used to learn hierarchical features in the data. Deep learning refers to the process of data (e.g., images) and corresponding labels (e.g., “carious tooth,” or “specific area on an image where a caries lesion is present”) being repetitively passed through the neural network during training, with the model parameters (so-called weights) being iteratively adjusted to improve the model's accuracy [1]. A deep learning-based convolutional neural network (CNN) algorithm considerably performed well in detecting dental caries in periapical radiographs [6]. It also successfully helped in detecting and classifying impacted supernumerary teeth in patients with fully erupted maxillary permanent incisors on panoramic radiographs [7]. The fully deep, fine-tuned mask R-CNN model performed well in automated tooth segmentation on panoramic images [8]. Additionally, it was also used for detecting apical lesions on panoramic radiographs [9].

Recently, an investigation showed that artificial neural networks (ANNs) could act as a second opinion to locate the apical foreman on radiographs and to enhance the accuracy of working length determination by radiography [10]. In another in vitro study, ANN also aided in the determination of shade, light-curing unit, and composite Vickers hardness ratio of bottom to top composites [11]. AI technology is found useful in assisting debonding probability of composite restorations in restorative dentistry [12].

Furthermore, an automated robotic system can fulfill the requirements of typical dental operations with accurate, safe, and three-dimensional (3D) tooth preparation [13, 14]. The AI convolutional neural network (CNN) can be utilized for classifying dental arches and designing removable partial dentures [15]. AI can analyze the impact of orthognathic treatment on facial attractiveness and age appearance. It offers a new feature that permits scoring of facial attractiveness and apparent age objectively and reproducibly [16]. Automated integration of facial and intraoral images of anterior teeth benefits dentists to analyze the shape and position of maxillary anterior teeth [17].

In a nutshell, the last decade has seen a surged with breakthrough in advancement of technology associated with artificial intelligence. However, it is still uncertain how information available in the literature regarding AI can assist in diagnosis, planning, and management of dental diseases. Therefore, to understand the current trends of AI in dentistry and its application, a systematic review was carried out on studies which have discussed different modalities of artificial intelligence, its application, and outcome in dentistry.

## 2. Materials and Methods

### 2.1. Focused Question

This systematic review was conducted using PRISMA (Preferred Reported Items for Systematic Review and Meta-analysis) guidelines. Our intended question was “Which artificial intelligence techniques are practiced in dentistry, and how AI is improving the diagnosis, clinical decision making, and outcome of dental treatment?” The question was constructed according to the Participants Intervention Comparison Outcome and Study (PICOS) strategy [18].

*Population*: patient/simulator faciodental images (two-dimensional image (2D), three-dimensional (3D), radiographs (periapical, bitewing, orthopantomography, and cone-beam computed tomography), CAD/CAM (computer-aided design and computer-aided manufacturing). Virtual dental models.

*Intervention*: AI techniques (deep learning, natural language processing, and robotics) applied in diagnosis, management, and predicting prognosis of dental treatment.

*Comparison*: automatic algorithm, testing models, image analysis, and rater opinions.

*Outcome*: analysis of AI performance, accuracy/precision, sensitivity, rating, CDS: clinical decision support, AUC: area under the curve, and AI applicability in different dental specialties.

*Study design type*: for this review, we considered both observational (case control and cohort) and interventional (trials) based studies, published in the English language.

### 2.2. Eligibility Criteria

The subsequent articles were reviewed for inclusion criteria: (1) original articles relevant to AI in dentistry, (2) clinical trials, (3) nonclinical trials, (4) observational studies, and (5) English language articles, whereas review articles, letters to editors, commentaries, grey literature, case reports, and articles with less than 10 participants or specimen were excluded.

### 2.3. Search Methodology

The medical subject heading (MeSH) terms are artificial intelligence (AI), dentistry, AI in dentistry, neural networks and dentistry, machine learning, AI dental imaging, and AI treatment recommendations; electronic search was carried out with PubMed/MEDLINE, ScienceDirect, Scopus, Web of Science, and Cochrane Collaboration databases. The articles published in the years 2000 to 2020 were targeted. The duration of data extraction was between 10 and 12 weeks. The last search was performed in the month of January 2020. Two calibrated reviewers (N.A. and W.Q.) performed the search. Disagreements and discrepancies were resolved by consensus, and a third examiner (F.Z.) was consulted. All the titles and abstracts were read thoroughly from the articles searched primarily, and nonrelevant studies were excluded. The relevant articles were enlisted and scrutinized for any similar studies which matched our inclusion criteria. For extraction of pertinent results, we read full texts of the included studies and the findings were recorded.

### 2.4. Quality Assessment of Included Studies

Quality assessment of included articles was carried out according to the standard parameters described in the Cochrane Handbook for Systematic Reviews of Interventions (v5.1.0) [19]. The parameters were patient randomization, blinding procedure, withdrawal/dropout reported; statistical analysis was used and stated clearly, execution of sample size estimation, multiple variables measurement, clear inclusion and exclusion criteria, comprehensible examiner reliability tested and clearly report all expected outcomes. The quality of each study was further classified into low, medium, and high risk of bias. The same 2 review authors autonomously sort out the search to amplify the number of studies recovered. The reviewers surveyed every selected article for the predefined consideration criteria and directed impartial appraisals, and any ambiguity was settled by discussion and agreement or by consultation with a third reviewer (F.Z.).

The Newcastle-Ottawa quality assessment scale (NOS) for case-control studies [20] was used for further analysis of the included articles. The analysis was based on the three core quality analysis parameters: case and group (selection, definition, and representativeness), comparability (comparison of case and control groups; analysis and control of confounding variable), and exposure (outcome assessment, i.e., analysis of golden percentage estimation in patients by different examiners; evaluation of study outcome related to different teeth measurements clinically; use of a universal assessment method for both control and case groups; dropout rate of patients in the included studies). A star system was adopted for rating the included studies. Each item in selection and outcome category received a maximum of 01 star while 02 stars were assigned for comparability if sufficiently reported. Each study total scored from 1 to 8 stars. Due to heterogeneity of the outcome and variables in selected studies, the research team was not able to conduct meta-analysis in the current review.

## 3. Results

### 3.1. Search Results

**Figure 3:**
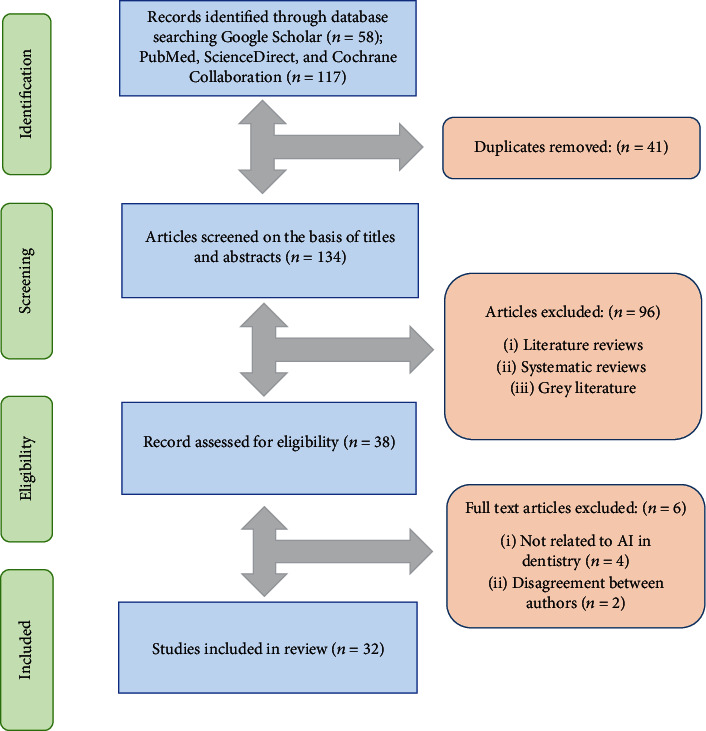
The PRISMA flow diagram for literature search performed in this study.

The primary search identified 175 articles based on key terms. Following those, 41 duplicates were removed, and 134 articles were screened based on title and abstracts. The search was further narrow down, and 96 irrelevant articles were excluded. The remaining 38 full-text articles were assessed for eligibility. Additionally, 6 full-text articles were further excluded. The 32 relevant articles were finally included and analyzed in the review. The PRISMA flow diagram for the literature search strategy is described in Figure 3. The excluded studies, in addition to their reasons for exclusion, are mentioned in Table 1.

### 3.2. General Characteristics of Included Studies

The general characteristics of the included studies are summarized in Table 2. The data were extracted from articles about the proposed study design: the authors' ID, year of publication, study and control groups, area of application in dentistry, assessment methods, follow-up period, and outcome of the study.

The included studies were ranged from the year 2000 to 2020. The studies were from four categories: cohort study [5, 16, 27], case control [28, 29], clinical trials [6–12, 17, 30–43], and experimental trials [13–15, 44, 45]. The follow-up period was mentioned in one study [43]. The various techniques of artificial intelligence was applied in the field oral and maxillofacial surgery [27–29, 38, 45], oral medicine [28], oral radiology [42], esthetic dentistry [17, 44], restorative dentistry [11–13, 34, 36, 40], endodontics [6, 9, 10, 41], oral diagnosis [5–9, 15, 33, 34, 36, 37, 42, 43, 45], orthodontics and orthognathic surgery [16, 35], forensic dentistry [8], gerodontology [39], implantology [43], periodontics [31, 32], and prosthodontics [14, 15, 30].

### 3.3. General Outcomes of Included Studies

The different modalities of artificial intelligence showed favorable outcomes. The deep learning with CNN's performed well in predicting the debonding probability of CAD/CAM crowns from 3D models [12], and it functioned considerably well in detecting apical lesion and dental caries in periapical (PA) and panoramic radiography [9, 36, 40, 42]. In addition to this, it has been proved to be accurate in predicting the treatment of dental decay based on radiographic images [6].

AI also has been proved to assist dentists in implant treatment starting from diagnosis to surgery by proficient and certain radiological evaluation [43]. Further, AI aided in the detection and classification of impacted supernumerary teeth, in the maxillary incisor region on periapical radiographs [7]. Along with AI, automation of tooth segmentation can be achieved through dental panoramic images [8].

CNN has been used to classify dental arches, and multilayer CNN also improves the radiographic diagnosis of proximal caries [33]. Machine learning computer algorithmic tool also facilitates detecting and classifying dental restoration in panoramic images [5]. ANN has been used to determine accurate working length on radiographs [10]. Likewise, a neural network and web-based system was able to assists in characterization of TMJ health and temporomandibular joint osteoarthritis (TMJOA) at clinical, imaging, and biological levels [29, 30, 38]. Furthermore, the computer color matching (CCM) technique provides an accurate color matching of dental restorations, together with the automatic laser ablation system for clinical crown preparation [14, 44]. Overall, the above methods if introduced into routine practice can be helpful in diagnosis and treating dental diseases.

### 3.4. Results of Quality Assessment

According to the standards described in the Cochrane Handbook for Systematic Reviews of Interventions (v5.1.0) [19], the following findings were recorded. Out of the 32 studies [5–17, 27–45] assessed, 1 study employed blinding [6]. In 5 studies, randomizations [5, 7, 34, 37, 39] were performed. The dropout rate was mentioned in 31 studies [5–11, 13–17, 27–45]. The study variables were analyzed for accuracy in 30 studies [5–13, 15–17, 27–29, 37, 39–45]. Sample size was mentioned in 31 studies [5–17, 27–33, 35–45]. The inclusion and exclusion criteria were clearly mentioned in 30 studies [5–15, 17, 27–37, 39–45]. The examiner reliability was also applied in 30 studies [5–13, 15–17, 27–31, 33–45]. Additionally, the outcome of study was prespecified in 28 studies [5–10, 13–17, 27–33, 35–37, 39–45]. The quality of 25 studies was rated as low [5–10, 13, 15, 17, 27–29, 31–33, 39–45, 33–35–37], whereas 7 studies were rated as having a moderate risk of biasness [11, 12, 14, 16, 30, 34, 38]. The quality assessment of the included studies is shown in Table 3.

Furthermore, “the quality assessment of selected studies on NOS [20] was ranging from 4 to 8 stars.” A mean score of 7 was achieved for the included studies, as mentioned in Table 4. Thirty-one studies fall in the moderate bias category [5–17, 27–29, 31–45] while 1 study had a high risk of biasness [30].

## 4. Discussion

The AI digital systems have unquestionably changed the direction of dentistry [46]. The AI modalities: machine learning, deep learning, cognitive computing, computer vision (recognizes the content in photos and videos), and natural language processing (to both analyze and generate human speech with the help of machines), are promising and practiced in dentistry [47]. Along the advent of AI better restoration, options are available with longer shelf life and superior esthetics and function [12, 13]. AI models are bringing greater efficiency and accuracy, capitalizing on the interest, capabilities, and skills of those involved [48]. Effective and efficient interprofessional and clinician-patient interactions have evolved using these new ways, with AI students have new ways of learning through research and the data collected can be efficiently utilized for forensic and epidemiological uses [49, 50]. Extensive research has been carried out on the application, benefits, and comparison of AI with human skills around the globe. The purpose of this systematic review was to investigate the quality and outcome of studies into artificial intelligence techniques, analysis, and its effect in dentistry.

Among the studies reviewed, it was revealed the application of artificial intelligence in dentistry is ample. Studies have found that the implications of AI in practice will facilitate dentists at every step. For instance, a neural network is beneficial in screening for oral cancer and precancer conditions, diagnosing bisphosphonate-related osteonecrosis before surgical removal of teeth and evaluating cervical lymph node metastasis of carcinoma after comparing it with magnetic resonance imaging [21, 28]. Furthermore, the computer color matching (CCM) technique provides an accurate color matching of dental restorations, together with the automatic laser ablation system for clinical crown preparation [14, 44]. The methodology used varied among the studies as to how the data were collected and analyzed and the AI technique developed. Therefore, a comparison of the studies was difficult.

The AI models suggested a positive impact in assisting dental diagnostics. Therefore, it can assist dentists in achieving correct interpretations of dental anomalies and minimizing human error. This review suggests that computer-based neural network plays a supporting role to dental practitioners, in decision making and minimizing errors during execution of dental treatment planning.

Furthermore, the current review proposes AI, a reliable technology for appraising the depth of dental caries, apical lesion diagnosis, working length determination, classification of dental arches, tooth segmentation, TMJ osteoarthritis, and early detection of early osteoporosis in jaws on panoramic radiographs [6, 8–10, 15, 36, 38, 45]. Rekow used a machine-learning algorithm to detect and classify dental restorations on panoramic images [25]. Kuwada et al. revealed that “DetectNet and AlexNet” appeared potentially useful in classifying the presence of impacted supernumerary teeth in the maxillary incisor region on panoramic radiographs [7]. Drevenstedt et al. used voice commands for recording patients' history and data, making suggestions during an ongoing dental procedure, scheduling patients' appointment, reminders for routine checkups, and necessary dental consultations [51]. The artificial neural network (ANN) models using bitewing photographs showed 97.1% accuracy for the dental caries diagnosis, 95.1% precision, a specificity of 94.3%, and a sensitivity ranging from 85% to 99.6% [42]. Sornam and Prabhakaran depicted an accuracy ranging from 85 to 100% using the AI model, “back-propagation neural networks” (BPNN) in dental caries classification [40]. However, comparisons among the studies were difficult because of differences in the methods used.

Despite the fact that the outcome of reviewed studies is auspicious, this study has few limitations. For example, the quality assessment of the literature conceded that there is a possibility of bias. The complexity of a particular system or mechanism, cost, and equipment of each setup need to be considered, including the training required for each AI model. Further research, exposure, and implementation are required. The worthwhile outcomes are not achieved yet due to the unavailability of accurate and sufficient data. In short, challenges exist both in technical and ethical aspects.

Nonetheless, in the future, the AI-based comprehensive care system will analyze big data including faciodental images and other records. AI models will provide reliable information and refined the clinical decision-making process. Infect AI is expected to establish high-quality patient care, innovative research, and state-of-the-art development in dentistry. Artificial intelligence and machine learning will aid to automation of aesthetic evaluation, smile design, and oral rehabilitation. By far, a change is not easy to adapt, but gradually, the application of AI in dental practice will become a necessity and might drive patient's demand too. AI has the proven ability to rationalize and take actions in the best manner of achieving a specific goal; this automated model can easily execute tasks, from simple to complex in nature.

## 5. Conclusions

At present, AI has been used vastly in dentistry. It has the potential to revolutionize oral health care by assisting in addressing the weaknesses grimly criticized in conventional dental care.

Based on the findings of this systematic review, it was concluded that

1. AI techniques assist dental practitioners in numerous ways, from decreasing the chairside time, saving extra steps, achieving excellent infection control, and providing quality treatment with accuracy and precision
2. AI can be successfully used for patient diagnosis, clinical decision making, and prediction of dental failures. Hence, it is a reliable modality for future application in oral diagnosis, oral and maxillofacial surgery, restorative dentistry, prosthodontics, orthodontics, endodontics, forensic dentistry, radiology, and periodontics
3. However, AI is increasing the scope of state-of-the-art models in dentistry but is still under development. Further studies are required to assess the clinical performance of AI techniques in dentistry

## Figures and Tables

**Table 1:** Methodological list of studies excluded from this review and reasons for exclusion (*n* = 5).

Author and year	Reason of exclusion
Gould [21] 2002	Disagreement between authors
Van der Meer et al. [22] 2016	Not AI, it was related to 3D printing guides
Vera et al. [23] 2013	AI-related to dental biotechnology
Leeson [24] 2020	Disagreement between authors
Rekow [25] 2020	Not AI, it was related to digital dentistry
McCracken et al. [26] 2000	Not AI, it was related to computer-assisted learning program

AI: artificial intelligence.

**Table 2:** Characteristics of selected studies (*n* = 33).

Author and year	Study design	Groups		Application	Assessment method	Follow-up period	Outcome
		Study	Control				
Abdalla-Aslan et al. [5] 2020	Cohort study	Machine learning computer vision algorithms	NA	OD	Automatic algorithm was used to detection and classification restoration while vector machine algorithm with error-correcting output codes was applied for cross-validation	NA	Machine learning demonstrated excellent performance in detecting and classifying dental restorations on panoramic images
Bouchahma et al. [6] 2019	Clinical trial	CNN	NM	OD and endodontics	Prediction of three types of treatments; fluoride, filling, and root canal treatments. The model was trained to learn on dataset of 200 X-ray images of patients' teeth collected	NM	DL overall accuracy was 87%. The best prediction was the fluoride treatment with 98%, followed by RCT detection 88% and filling 77%
Kuwada et al. [7] 2020	Clinical trial	DetectNet, AlexNet, and VGG-16	NM	OD	400 images were randomly selected as training data, and 100 as validating and testing data. The remaining 50 images were used as new testing data. Recall, precision, and F-measure were used for detection of impacted teeth	NM	DetectNet and AlexNet appear to have potential use in classifying the presence of impacted supernumerary teeth in the maxillary incisor region on PR, while VGG-16 showed lower values
Lee et al. [8] 2020	Clinical trial	CNN on 20 automated 20 tooth segments	Oral radiologist manually performed individual tooth annotation on the PA	OD and forensic dentistry	846 images with tooth annotations from 30 PA were used for training, and 20 as the validation and test sets. A fully deep learning method using the mask R-CNN model was implemented through a fine-tuning process to detect and localize the tooth structures	NM	It achieved high performance for automation of tooth segmentation on dental panoramic images. The proposed method might be applied in the first step of diagnosis automation and in forensic identification
Ekert et al. [9] 2019	Clinical trial	CNN to detect AL	Six independent examiners detect AL	Endodontics and OD	NN was trained and validated via 10 times repeated group shuffling. Results were compared with the majority vote of 6 examiners who detected ALs on an ordinal scale	NM	A moderately deep CNN showed satisfying discriminatory ability to detect ALs on panoramic radiographs
Saghiri et al. [10] 2012	Clinical trial	ANN	Endodontist's opinion	Endodontics	Working length was determined and confirmed radiographically by endodontists and compared with ANN, and stereomicroscope as a gold standard after tooth extraction in cadaver	NM	ANN was more accurate than endodontists' determinations when compared with measurements by using the stereomicroscope
Arisu et al. [11] 2018	Clinical trial	ANN	NM	Restorative dentistry	Obtained measurements and data were fed to an ANN to establish the correlation between the inputs; composite shade curing units and outputs; tooth number	NM	ANN showed that the light-curing units and composite parameter had the most significant effect on the bottom to top Vickers hardness ratio of the composites
Yamaguchi et al. [12] 2019	Clinical trial	12 dislodge CAD/CAM composite resin crowns with DL	12 trouble-free CAD/CAM composite resin crowns	Restorative dentistry	Convolution neural network (CNN) technique was used to predict debonding of composite crowns using 2D images captured from 3D stereolithography models	NM	Deep learning with CNN model showed good performance in terms of dislodgement predictability of composite crowns through 3D stereolithography models
Otani et al. [13] 2015	Experimental study	Ten veneer preparation with a robotic arm	Ten conventional veneers prepared by a clinician	Restorative dentistry	Accuracy and precision of veneer preparation were compared for all sites and separately for each tooth surface (facial, finish line, incisal) through 3D images and computation	NM	The robotic arm was able to prepare the tooth model as accurately as the control. However, a better finish line accuracy and precision was showed by the robotic arm
Wang et al. [14] 2014	Experimental study	Automatic laser ablation system for tooth crown preparation	NM	Prosthodontics	A layer-by-layer ablation method is developed to control the laser focus during the crown preparation	NM	The movement range and the resolution of the robotic system meet the satisfying requirements of typical dental operations for clinical crown preparation
Takahashi et al. [15] 2020	Experimental study	CNN	NM	Prosthodontics and OD	1184 images of dental arches were classified into four arch types. A CNN method to classify images was developed using tensor flow and Kera's deep learning libraries	NM	The results of this study suggest that dental arches can be classified and predicted using a CNN
Patcas et al. [16] 2019	Cohort study	CNN was applied in posttreatment photographs of 146 orthognathic patients	Pretreatment photographs of 146 patients	Orthodontics	CNN-based technique was used to compare facial attractiveness and apparent age of patients through pre- and posttreatment photographs	NA	Artificial intelligence can be used to detect facial attractiveness scores and apparent age in orthognathic surgery patients
Li et al. [17] 2020	Clinical trial	50 oral images and 274 anterior through automated photo integrating system	Manual segmentation system	Esthetic dentistry	The facial and intraoral key points were detected by an automatic algorithm and compared with manual segmentation on standard photographs	NM	The proposed automated system can eliminate the need for dentists to employ a laborious image integration process and has potential for broad applicability in the field of esthetic dentistry
Li et al. [44] 2015	Experimental study	BPNN and GA neural network	Traditional neural network	Esthetic dentistry	The weighs and threshold values of GA and BPNN were compared for assistance in tooth color matching in dentistry	NM	GA and BP have practical application and can make teeth color matching objective and accurate
Edinger [30] 2004	Clinical trial	ROSY, a robot-like electronic simulator	NM	Prosthodontics	Accuracy of the simulator was measured for all directions in space by registering eccentric jaw positions on both sides of 10 subjects	NM	Its accuracy may render it suitable for clinical applications
Meissner et al. [31] 2006	Clinical trial	Automated smart ultrasonic calculus detection system	NM	Periodontics	The detection device is based on a conventional dental piezoelectric ultrasonic hand piece with a conventional scaler insert	NM	It was able to distinguish between different tooth surfaces in vitro independently from tip movements
Meissner et al. [32] 2005	Clinical trial	A novel calculus recognition device applied on 70 extracted teeth	NM	Periodontics	Impulse generator, coupled to a conventional piezo-driven ultrasonic scaler, sends signals to the cementum via the tip of an ultrasound device	NM	This system is able to function correctly, independent of the lateral forces and the tip angle of the instrument
Devito et al. [33] 2008	Clinical trial	Multilayer perceptron neural network	Twenty-five dental specialists with 20 years' experience	OD	Evaluation of proximal caries on radiographic through ANN	NM	AI improves the radiographic diagnosis of proximal caries by 39.4%
Kositbowornchai et al. [34] 2006	Clinical trial	Learning vector quantization (LQV, NN)	NM	Restorative dentistry and OD	Tooth sections and microscopic examinations were used to confirm the actual dental caries status	NM	AI plays a useful and supporting in making dental caries diagnosis
Patcas et al. [35] 2019	Clinical trial	Ten images evaluated by CNN model	Ten images were analyzed by laypeople, orthodontists, and oral surgeon on a visual analogue scale	Orthodontics	Decision on profile and frontal images of cleft patients were compared between CNN technique and conventional rater group to evaluate facial attractiveness	NM	AI can be a helpful tool to describe facial attractiveness and overall analysis were comparable with the rater groups
Lee et al. [36] 2018	Clinical trial	CNN	Four calibrated board-certified dentists	OD and restorative dentistry	A pretrained GoogleNet Inception v3 CNN network was used for preprocessing and transfer learning	NM	CNN provides considerably good performance in detecting dental caries in PR
Vranckx et al. [37] 2020	Clinical trial	CNN and ResNet-101	Manual measurements by 2 observers	OD	CNN and ResNet-101 jointly predicted the molar segmentation maps and an estimate of the orientation lines	NM	Fast, accurate, and consistent automated measurement of molar angulations on dental PR
Lee et al. [38] 2020	Clinical trial	Fifty cases of class2 TMJOA	Fifty cases of normal TMJ	OMFS	The condylar head was classified into 2 categories and tested by making 300 images	NM	AI can be used to support clinicians with diagnosis and decision making for treatments of TMJOA
Hung et al. [39] 2019	Clinical study	Machine learning method ANN was used on bitewing radiograph	Training group consisting of conventional radiograph analysis	Gerontology	Support vector machine (ANN) was used to detect root caries on radiograph by determining AUC	NM	Support vector machine showed 97.1% accuracy, 95.1% precision, 99.6% sensitivity, and 94.3% specificity for root caries detection
Cui et al. [27] 2020	Cohort study	CDS model applied to 3559 patient records	Two prosthodontists' opinion	OMFS	CDS model was used to predict the outcome of teeth extraction through electronic dental records	NA	The machine learning CDS was an efficient tool to predict teeth extraction outcome
Sornam and Prabhakaran [40] 2019	Clinical study	LB-ABC with BPNN	BPNN classifier	Restorative dentistry	The BPNN classifier is compared with the LB-ABC-based BPNN classifier for dental caries classification	NM	The learning rate generated by the LB-ABC for the BPNN classifier achieved the best training and testing accuracy of 99.16%
Setzer et al. [41] 2020	Clinical study	Evaluation of periapical lesion by DL method	Rating by OMF radiologist, an endodontist, and a senior graduate student	Endodontics	The CBCT segmentation was assessed by DL, CNN detection	NM	DL algorithm trained in a limited CBCT environment showed excellent results in lesion detection accuracy
Cantu et al. [42] 2020	Clinical study	Caries detection on bitewing radiograph with DL	Opinion of four experienced dentists	OD, OR	CNN (U-Net) and Intersection-over-Union were used to detect caries on radiographs	NM	The deep neural network was accurate than dentists
Aliaga et al. [45] 2020	Experimental study	Automatic computation and intelligent image segmentation of 370 radiographs	Expert dentist opinion	OD, OMFS	Automatic computation for analysis of mandibular indices and osteoporosis detection	NM	Automatic computation of mandibular indices and intelligent image segmentation was an efficient and reliable approach for early osteoporosis detection
Kim et al. [28] 2018	Case-control study	Machine learning prediction models for BRONJ after extraction in 125 patients with drug use	Conventional methods, serum CTX level	OMFS/OM	Five machine learning methods such as logistic regression model, decision tree, support vector machine, ANN, and random forest were applied to predict BRONJ at extraction sites	NA	Machine learning showed superior performance in predicting BRONJ compared with serum CTX level and drug holiday period
Dumast et al. [29] 2018	Case-control study	17 tested OA subjects evaluated with deep CNN on 3D images	17 age and sex-matched control subjects without OA	OMFS	Deep neural network classifier of 3D condylar morphology (ShapeVariationAnalyzer, SVA), and a flexible web-based system for data storage, computation and integration (DSCI) of high dimensional imaging, clinical, and biological data	NA	Deep neural network is a useful tool for classification of TMJOA
Sorkhabi and Khajeh [43] 2019	Clinical trial	3D deep CNN and CBCT	Postextraction clinical parameter measurements	OD and implant dentistry	3D CNN method was used to measure alveolar bone density on CBCT images	6 months	3D deep CNN technique can accurately classify alveolar bone. Pattern, which is helpful in dental implant placement and diagnosis

NA: not applicable; NM: not mentioned; OMFS: oral and maxillofacial surgery; OM: oral medicine; OP: oral pathology; OR: oral radiology; OD: oral diagnosis; AL: apical lesion; CNN: convolutional neural networks; ANN: artificial neural networks; 3D: three dimensional; DL: deep learning; CAL: computer-assisted learning; CAD/CAM: computer-aided design/computer-aided manufacturing; 2D: two dimensional; TMJOA: temporomandibular joint osteoarthritis; OA: osteoarthritis; BPNN: back-propagation neural networks; CDS: clinical decision support systems; BRONJ: bisphosphonate-related osteonecrosis of the jaw; LB-ABC: logit-based artificial bee colony optimization algorithm; VGG-16: Visual Geometry Group; PA: periapical radiograph; CBCT: cone-beam computerized tomography; GA: genetic algorithm; serum CTX: serum C-terminal telopeptide; AUC: area under the curve.

**Table 3:** Methodological quality assessment results of the included studies (*n* = 33).

Author and year	Randomization	Blinding	Withdrawal/dropout mentioned	Variables measured many times	Sample size estimation	Inclusion/exclusion criteria clear	Examiner reliability tested	Expected outcomes prespecified	Quality of study/bias risk
Abdalla-Aslan et al. [5] 2020	Yes	No	Yes	Yes	Yes	Yes	Yes	Yes	Low
Bouchahma et al. [6] 2019	No	Yes	Yes	Yes	Yes	Yes	Yes	Yes	Low
Kuwada et al. [7] 2020	Yes	No	Yes	Yes	Yes	Yes	Yes	Yes	Low
Lee et al. [8] 2020	No	No	Yes	Yes	Yes	Yes	Yes	Yes	Low
Ekert et al. [9] 2019	No	No	Yes	Yes	Yes	Yes	Yes	Yes	Low
Saghiri et al. [10] 2012	No	No	Yes	Yes	Yes	Yes	Yes	Yes	Low
Arisu et al. [11] 2018	No	No	Yes	Yes	Yes	Yes	Yes	No	Moderate
Yamaguchi et al. [12] 2019	No	No	Unclear	Yes	Yes	Yes	Yes	No	Moderate
Otani et al. [13] 2015	No	No	Yes	Yes	Yes	Yes	Yes	Yes	Low
Wang et al. [14] 2014	Unclear	Unclear	Yes	No	Yes	Yes	No	Yes	Moderate
Takahashi et al. [15] 2020	No	No	Yes	Yes	Yes	Yes	Yes	Yes	Low
Patcas et al. [16] 2019	No	No	Yes	Yes	Yes	Unclear	Yes	Yes	Moderate
Li et al. [17] 2020	No	No	Yes	Yes	Yes	Yes	Yes	Yes	Low
Li et al. [44] 2015	No	No	Yes	Yes	Yes	Yes	Yes	Yes	Low
Edinger [30] 2004	Unclear	Unclear	Yes	Yes	Yes	Yes	No	Yes	Moderate
Meissner et al. [31] 2006	No	No	Yes	Yes	Yes	Yes	Yes	Yes	Low
Meissner et al. [32] 2005	No	No	Yes	Yes	Yes	Yes	Yes	Yes	Low
Devito et al. [33] 2008	No	No	Yes	Yes	Yes	Yes	Yes	Yes	Low
Kositbowornchai et al. [34] 2006	Yes	No	Yes	Yes	No	Yes	Yes	No	Moderate
Patcas et al. [35] 2019	No	No	Yes	Yes	Yes	Yes	Yes	Yes	Low
Lee et al. [36] 2018	No	No	Yes	Yes	Yes	Yes	Yes	Yes	Low
Vranckx et al. [37] 2020	Yes	No	Yes	Yes	Yes	Yes	Yes	Yes	Low
Lee et al. [38] 2020	No	No	Yes	No	Yes	No	Yes	No	Moderate
Hung et al. [39] 2019	Yes	No	Yes	Yes	Yes	Yes	Yes	Yes	Low
Cui et al. [27] 2020	No	No	Yes	Yes	Yes	Yes	Yes	Yes	Low
Sornam and Prabhakaran [40] 2019	No	No	Yes	Yes	Yes	Yes	Yes	Yes	Low
Setzer et al. [41] 2020	No	No	Yes	Yes	Yes	Yes	Yes	Yes	Low
Cantu et al. [42] 2020	No	No	Yes	Yes	Yes	Yes	Yes	Yes	Low
Aliaga et al. [45] 2020	No	No	Yes	Yes	Yes	Yes	Yes	Yes	Low
Kim et al. [28] 2018	No	No	Yes	Yes	Yes	Yes	Yes	Yes	Low
Dumast et al. [29] 2018	No	No	Yes	Yes	Yes	Yes	Yes	Yes	Low
Sorkhabi and Khajeh [43] 2019	No	No	Yes	Yes	Yes	Yes	Yes	Yes	Low

^∗^A study was graded to have a low risk of bias if it yielded 6 or more “yes” answers to the 9 questions, moderate risk if it yielded 3 to 5 “yes” answers, and high risk if it yielded 2 “yes” answers or less.

**Table 4:** Newcastle-Ottawa scale based quality assessment of selected studies (*n* = 33).

Author and year	Selection	Compatibility	Exposure	Newcastle-Ottawa quality (total)
Abdalla-Aslan et al. [5] 2020	∗∗∗	∗	∗∗∗	7
Bouchahma et al. [6] 2019	∗∗∗∗	∗	∗∗∗	7
Kuwada et al. [7] 2020	∗∗∗	∗	∗∗∗	7
Lee et al. [8] 2020	∗∗∗	∗	∗∗	6
Ekert et al. [9] 2019	∗∗∗	∗	∗∗∗∗	8
Saghiri et al. [10] 2012	∗∗∗	∗	∗∗∗	7
Arisu et al. [11] 2018	∗∗	∗	∗∗∗	6
Yamaguchi et al. [12] 2019	∗∗∗	∗	∗∗∗∗	8
Otani et al. [13] 2015	∗∗	∗∗	∗∗∗	7
Wang et al. [14] 2014	∗∗	∗	∗∗∗	6
Takahashi et al. [15] 2020	∗∗∗	∗∗	∗∗	7
Patcas et al. [16] 2019	∗∗∗	∗	∗∗	6
Li et al. [17] 2020	∗∗∗∗	∗	∗∗∗	8
Li et al. [44] 2015	∗∗∗	∗	∗∗∗∗	8
Edinger [30] 2004	∗	∗	∗∗	4
Meissner et al. [31] 2006	∗∗	∗	∗∗∗	6
Meissner et al. [32] 2005	∗∗∗	∗	∗∗	6
Devito et al. [33] 2008	∗∗∗	∗	∗∗∗∗	7
Kositbowornchai et al. [34] 2006	∗∗∗	∗	∗∗	6
Patcas et al. [35] 2019	∗∗∗	∗	∗∗∗	7
Lee et al. [36] 2018	∗∗∗	∗	∗∗	6
Vranckx et al. [37] 2020	∗∗∗	∗	∗∗∗	7
Lee et al. [38] 2020	∗∗	∗	∗∗∗	8
Hung et al. [39] 2019	∗∗∗	∗	∗∗∗	7
Cui et al. [27] 2020	∗∗∗	∗	∗∗	6
Sornam and Prabhakaran [40] 2019	∗∗∗	∗	∗∗∗	7
Setzer et al. [41] 2020	∗∗∗	∗	∗∗∗	7
Cantu et al. [42] 2020	∗∗∗∗	∗	∗∗∗	8
Aliaga et al. [45] 2020	∗∗∗	∗	∗∗	6
Kim et al. [28] 2018	∗∗∗	∗	∗∗∗	7
Dumast et al. [29] 2018	∗∗∗	∗	∗∗∗	7
Sorkhabi and Khajeh [43] 2019	∗∗∗	∗	∗∗∗	7

^∗^A study can be awarded a maximum of 1 star for each numbered item within the selection and exposure categories. A maximum of 2 stars can be given for comparability. Each study can be awarded a total of 9 stars. A study was rated to have a low risk of biasness if it received the maximum allowed number of 9 “stars” while moderate risk if it received 8, 7, or 6 “stars” and high risk if it received 5 “stars” or less.

## Data Availability

The raw data used to support the findings of this study are included within the article.
